# PI3K and AKT: Unfaithful Partners in Cancer

**DOI:** 10.3390/ijms160921138

**Published:** 2015-09-03

**Authors:** Seraina Faes, Olivier Dormond

**Affiliations:** Department of Visceral Surgery, Centre Hospitalier Universitaire Vaudois and University of Lausanne, Pavillon 4, Av. de Beaumont, Lausanne 1011, Switzerland; E-Mail: seraina.faes@chuv.ch

**Keywords:** cancer, signaling, PI3K, AKT, therapies

## Abstract

The phosphatidylinositol 3-kinase (PI3K)/AKT signaling pathway regulates multiple cellular processes. An overactivation of the pathway is frequently present in human malignancies and plays a key role in cancer progression. Hence, its inhibition has become a promising approach in cancer therapy. However, the development of resistances, such as the abrogation of negative feedback mechanisms or the activation of other proliferative signaling pathways, has considerably limited the anticancer efficacy of PI3K/AKT inhibitors. In addition, emerging evidence points out that although AKT is acknowledged as the major downstream effector of PI3K, both PI3K and AKT can operate independently of each other in cancer, revealing another level of complexity in this pathway. Here, we highlight the complex relationship between PI3K and AKT in cancer and further discuss the consequences of this relationship for cancer therapy.

## 1. Introduction

Targeting signaling pathways that are deregulated in human cancer has been a promising approach in cancer therapy [1]. The initial success of imatinib in chronic leukemia has validated this approach and has encouraged the development of additional therapies [2]. To date, a large number of new drugs that target mutated proteins responsible for tumor growth has already been tested in clinical trials. For example vemurafenib, a drug that targets the mutated version of B-Raf (v-raf murine sarcoma viral oncogene homologe B1) has been demonstrated to prolong survival in metastatic melanoma [3]. This represents a remarkable achievement, given the fact that advanced melanoma poorly respond to conventional treatments. Nevertheless, if targeted therapies prolong survival in advanced cancer patients, they do not cure cancer. Several drawbacks have been identified, the most important probably being the development of resistance by cancer cells and the tumor microenvironment [4].

The phosphatidylinositol 3-kinase (PI3K)/AKT signaling pathway is frequently deregulated in cancer and accordingly represents an important anticancer target [5]. Indeed, the PI3K/AKT signaling pathway plays a major role in regulating cellular processes that are features of cancer such as cell proliferation, survival or migration. In addition, activating mutations of these enzymes are also frequently observed in human cancer, leading to cancer growth. Thus, over the last years, drugs that target PI3K or AKT have been extensively developed and are being tested in clinical trials (Supplementary Tables S1 and S2) [6,7]. However, similarly to what is observed for other targeted therapies, adaptive resistance limits the antitumor efficacy of these drugs. It is thus important to fully characterize the interactions between the components of the pathway in order to generate new concepts that can be exploited therapeutically.

Over the last years, the use of drugs that target PI3K or AKT helped to understand the complexity of the biological consequences of blocking PI3K or AKT. In particular, emerging evidence has demonstrated that PI3K and AKT can act independently of each other, thus challenging the paradigm that PI3K and AKT closely interact to induce cellular transformation [8,9]. Here, after describing the major features of this pathway in cancer, we will review the complex relationship between PI3K and AKT in cancer and its relevance for cancer therapy.

## 2. Classes of PI3K Enzymes

The family of lipid kinases named phosphatidylinositol 3-kinase was discovered in the mid-1980s and subsequently shown to play a major role in cellular functions such as cell proliferation and survival [10,11]. PI3Ks mostly generate phospholipids serving to transduce signals generated by receptor tyrosine kinases and G protein-coupled receptors. According to their structure and substrate specificity, PI3Ks belong to three distinct categories called Class I, II and III [12,13]. Class I is further divided into Class IA and IB. Among the different classes of PI3K, Class IA PI3K seems to play the predominant role in cancer. These PI3Ks are formed by a catalytic p110 subunit as well as a regulatory p85 subunit. Whereas p110 generates phosphatidylinositol 3,4,5-triphosphate (PIP3), p85 is responsible for the interaction of PI3K with the upstream effectors. There are three different p110 isoforms: p110α and p110β, both being ubiquitously expressed, and p110δ whose expression is largely limited to the immune system [14]. Furthermore, distinct p85 exist in mammals termed p85α (and its splice variants p55α and p50α), p85β and p55γ. Activation of Class IA enzymes mostly occurs through tyrosine kinase receptors and certain oncogenes such as RAS (rat sarcoma) [12]. In addition, p110β and p110δ can also be activated by G protein-coupled receptors. Class IB PI3Ks are composed of a catalytic subunit p110γ that, unlike the other catalytic Class IA subunits, does not bind to the regulatory p85 subunit but instead to regulatory subunits p101 or p87. As a consequence, Class IB enzymes are exclusively regulated by G protein-coupled receptors and not by tyrosine kinase receptors. Moreover, their expression is more limited, as p110γ is mainly found in leukocytes as well as in the heart, liver, skeletal muscle and pancreas [12]. Both Class IA and IB phosphorylate phosphatidylinositol 4,5-biphosphate to generate PIP3. Class II PI3K are formed by only one catalytic protein existing in three different isoforms PI3KCA2α, PI3KCA2β and PI3KCA2γ. Unlike Class I enzymes, the cellular function of Class II PI3K remains poorly characterized [15]. Of note, these enzymes use phosphatidylinositol-4-phosphate as a substrate. Finally, similarly to Class II, Class III PI3K have one single catalytic subunit called VPS34 (vesicle-mediated vacuolar protein sorting 34) and are implicated in autophagy [16].

## 3. Downstream of Class I PI3K: AKT a Close Partner

**Figure 1:**
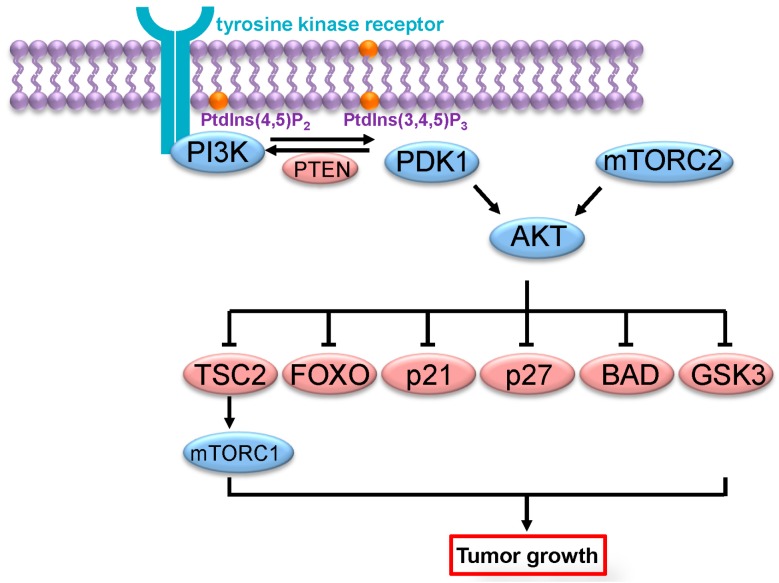
PI3K/AKT signaling pathway. Following its activation by tyrosine kinase receptors, PI3K catalyzes the phosphorylation of PtdIns(4,5)P_2_ (phosphatidylinositol 4,5-bisphosphate) to PtdIns(3,4,5)P_3_ (phosphatidylinositol 3,4,5-trisphosphate), resulting in the recruitment of PDK1 and AKT to the plasma membrane and their activation. AKT then acts upon multiple downstream effectors, leading to cell growth, proliferation and survival.

The Class I PI3K signaling pathway has been extensively characterized. Following activation, Class I PI3Ks generate PIP3 at the plasma membrane, which will induce the recruitment of proteins that have a pleckstrin homology (PH) domain such as PDK1 (pyruvate dehydrogenase lipoamide kinase isozyme 1) and AKT (Figure 1) [17]. Of note, PIP3 levels are tightly regulated by the activity of the tumor suppressor PTEN (phosphatase and tensin homolog), which by converting PIP3 back to phosphatidylinositol 4,5-bisphosphate exerts an opposing effect to PI3Ks [18]. Recruitment of PDK1 and AKT to the plasma membrane leads to the interaction of both kinases where PDK1 phosphorylates and partially activates AKT on a T308 residue [19,20]. Activation of AKT further requires an additional phosphorylation on S473 by mTORC2 (mechanistic target of rapamycin complex 2) [21]. Following activation, AKT phosphorylates a large number of downstream effectors including MDM2 (mouse double minute 2 homolog), GSK3β (glycogen synthase kinase 3 beta), FOXO (Forkhead box O transcription factor), BAD (Bcl-2-associated death promoter), Caspase-9, p27, PRAS40 (proline-rich AKT substrate of 40 kDa) and TSC2 (tuberous sclerosis complex 2), resulting ultimately in cell growth, survival and proliferation (Figure 1) [22]. To date, three different AKTs, (AKT1, AKT2 and AKT3), products of three different genes and sharing 80% amino acids homology, have been characterized. Some evidence points out that these isoforms have differential effects. Whereas AKT1 promotes growth and survival, AKT2 controls cellular invasiveness and mesenchymal characteristics [23,24].

Besides AKT and PDK1, other PH domain containing proteins were shown to be regulated by PIP3. These include guanine nucleotide exchange factors for the small GTPases RAC (RAS-related C3 botulinum toxin substrate), RAS and Rho and ADP-ribosylation factor families [11]. Since around 1700 proteins have been found to possess a PH domain, it becomes apparent that other proteins will in the future be identified as being part of the Class IA signaling pathway. Of note, only a small fraction of PH domains interact with PIP3, therefore it is not expected that all 1700 proteins will participate in the pathway [11]. Another level of complexity in the Class IA signaling pathway is the observation that at least two other protein modules distinct from the PH domain also interact with PIP3. Indeed, FYVE (domain identified in Fab1p) and PX (Phox homology) domains were both shown to bind PIP3. Future studies will help identify proteins with a domain that is regulated by PIP3 [11].

## 4. PI3K/AKT and Cancer

The role of PI3K in cancer was highlighted by the observation that the gene coding the catalytic α subunit of p110 is frequently mutated in the most common human cancers [25]. Two major mutations were found, one on exon 20 (H1047R) which may constitutively activate the enzyme and one on exon 9 (E545K) which blocks the inhibitory effects of the regulatory subunit p85 on p110α [26,27]. Furthermore, the tumorigenic potential of these mutations was proven in experimental settings [28]. In contrast, no oncogenic mutations were found in the other catalytic subunits p110β, p110γ and p110δ. An upregulation of these subunits has, however, been noted in various cancers and an overexpression of wild-type p110β, p110γ or p110δ leads to cell transformation in culture [29]. In addition to mutations of p110α, mutations of the regulatory p85α have also been reported in glioblastoma, ovarian cancer and colorectal cancer. The majority of these mutations are located in the interacting domain of p85α with p110, leading to the disruption of the inhibitory effect of p85α on p110, thus leading to constitutive PI3K signaling [30,31]. Finally aberrant PI3K signaling is also observed in cancers that have mutated or amplified receptor tyrosine kinases [32]. Since PI3K are direct effectors of these kinases, PI3K activity is constitutively activated in the absence of PI3K mutations. This is also true for activating mutations of RAS, a small GTPase frequently mutated in cancer. In this case, RAS can directly bind to p110 and induce its PI3K signaling [33]. In addition, loss of function of PTEN leads also to accumulation of PIP3 and activation of the pathway [34].

Similarly to PI3K, mutations in AKT family genes have been identified in human cancers. For instance, a single amino acid substitution, E17K, in the PH domain leads to a constitutive recruitment of AKT to the membrane [35]. Mutations of the kinase domain or amplification of the AKT2 have also been reported [36].

## 5. PI3K and AKT, not as Close in Cancer

Although AKT is viewed as a major downstream effector of PI3K, at least in physiological processes, several studies suggest that PI3K and AKT act independently of each other in cancers. For instance, the analysis of 547 human breast cancers showed no correlation between AKT phosphorylation and activating PI3K mutations. In contrast, AKT phosphorylation was significantly higher in tumors that expressed low level of PTEN [37]. Consistent with these observations, it was reported that AKT phosphorylation could be markedly reduced in tumor cell lines harboring PI3K activating mutations [38]. The latter suggests that an activation of PI3K not necessarily induces an increased activation of AKT in cancer cells. Furthermore, overexpression of PDK1 in human breast cancer cell lines increased anchorage independent growth and tumor formation, which was not prevented by AKT inhibition. Similarly, reduced anchorage independent growth of cancer cell following downregulation of PDK1 was not rescued by the expression of a constitutively active form of AKT [39]. Likewise, no correlation was found between the phosphorylation of AKT and the mutation status of PI3K in colon cancer cell lines [40]. Conversely, AKT activity can still be detected in cells treated with PI3K inhibitors [41,42]. Whereas AKT phosphorylation is inhibited by short-term treatment of cancer cells with PI3K inhibitors, prolonged treatment failed to block AKT phosphorylation suggesting that under chronic PI3K inhibition, other kinases are responsible for AKT activation [43]. Consistent with this observation, AKT activity and phosphorylation was still present despite ablation of PDK1, further highlighting possible alternative mechanisms than PI3K/PDK1 for AKT activation [44].

As mentioned previously, PIP3 levels and thus AKT activity are also regulated by PTEN, representing another level of complexity in the PI3K/AKT partnership. This is of importance, as in some cancers, PI3K and PTEN mutations were reported to be mutually exclusive [45]. Interestingly, activation of PI3K signaling either by PTEN loss or PI3K activation might result in different outcomes. For instance, breast cancer cells were more sensitive to PI3K inhibitors when PTEN was lost compared to the presence of activating mutations of PI3K [37]. Moreover, in a mouse model, targeting AKT was highly effective in blocking tumor growth of PI3K mutated melanoma whereas it had no effect on PTEN deficient melanoma [46]. Therefore, genetic alterations of PTEN and PI3K are not redundant. Interestingly, it was proposed that in tumors in which PI3K and PTEN mutations co-occur, sufficient levels of PIP3 are generated, resulting in a robust AKT signaling. In contrast, PI3K mutations alone may transduce AKT independent signals, and in this case, tumor growth depends on other downstream effectors than AKT [38].

## 6. Other Partners for PI3K in Cancer Development

**Figure 2:**
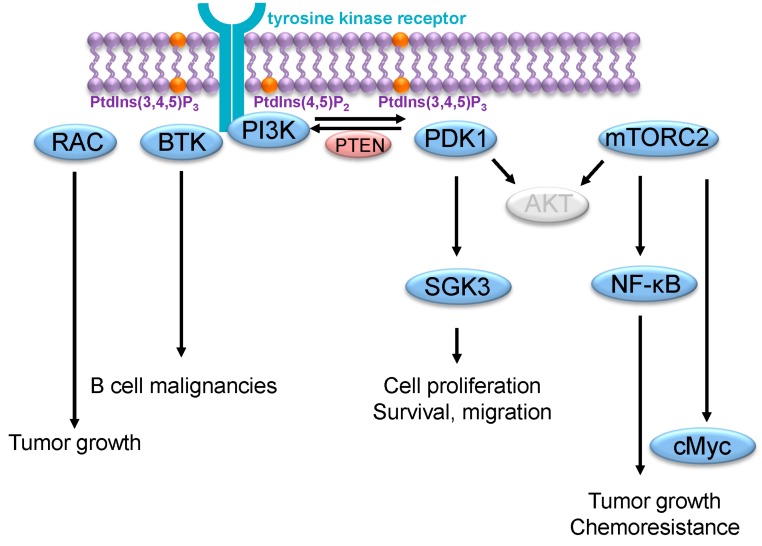
AKT-independent PI3K signaling in cancer. Several AKT-independent mechanisms used by PI3K to promote cancer growth have been described. They include activation of RAC, BTK in B cell malignancies, PDK1/SGK3 and mTORC2/NF-κB; mTORC2/c-Myc. Light gray ovals imply components of the signaling pathway that are not activated or bypassed.

Several studies tried to identify kinases other than AKT that are responsible for transformation in cancer cells harboring PI3K mutations (Figure 2). In this context, PDK1 represents an obvious candidate [47], as oncogenic functions of PDK1 through substrates other than AKT such as MAPK (mitogen-activated protein kinase) or PKCα (protein kinase C alpha) have already been reported [48,49]. More recently, emerging evidence has shown the importance of the SGK3 (serum- and glucocorticoid-inducible protein kinase 3) downstream of PDK1 in this process [9]. Using an shRNA screening targeting primarily kinases and phosphatases, SGK3 was identified as an important downstream mediator in breast cancer cells with PI3K mutations and low AKT phosphorylation levels [38]. In this context, GSK3 activation is mediated by the phosphoinositide phosphatase INPP4B (inositol polyphosphate-4-phosphatase type II B) whose expression enhances GSK3 activity while reduces AKT activity [50]. Interestingly, like AKT, SGK3 and two other isoforms, SGK1 and SGK2, belong to the AGC protein kinase family and are activated following their phosphorylation by PDK1 and mTORC2. AKT and SGK phosphorylate the same consensus substrate motif and therefore also possess similar substrates [51].

Besides PDK1, mTORC2 represents another kinase that might be involved in AKT-independent effects of PI3K signaling in cancer. mTORC2 activity relies on PI3K and was shown to be involved in cancer growth in several experimental models [52,53]. Although strong evidence emphasizes the importance of the phosphorylation and activation of AKT by mTORC2, recent studies have revealed AKT-independent pro-tumorigenic features of mTORC2. For example, in glioblastoma, epidermal growth factor receptor mutation induces mTORC2 activation, which results in proliferation and chemoresistance in an NF-κB (nuclear factor kappa B) dependent but AKT independent manner [54]. Similarly, an mTORC2/SGK1/NDRG1 (n-Myc downstream-regulated gene 1) signaling pathway mediates resistance to alkylating agents in glioma [55]. Furthermore, also in glioblastoma, mTORC2 controls glycolytic metabolism by the up-regulation of c-Myc (cellular myelocytomatosis oncogene) and independently of AKT [56]. In addition, in breast cancer cells, cell survival following DNA damage depends on mTORC2-mediated Chk1 (checkpoint kinase 1) activation [57].

RAC proteins are a subfamily of Rho family of small GTPases. RAC are active when GTP-bound, which allows them to interact and activate their target proteins. It is well established that PI3K activate RAC through several different mechanisms [58]. The role of RAC in tumorigenesis is complex and involves cell–cell adhesion, cell–matrix interaction, cell proliferation and cell survival [59]. In breast cancer cells, RAC is responsible for PI3K mediated MAPK activation [60].

The Burton’s tyrosine kinase (BTK) is another PH-domain containing protein that lies downstream of PI3K and whose cellular functions do not rely on AKT [61]. BTK is a key component of the B cell receptor signaling and accordingly functions as an important regulator of cell proliferation and survival in various B cell malignancies. Since its expression profile is limited to B cells, its activity mainly depends on PI3Kδ [62]. Interestingly, drugs that target PI3Kδ or BTK have shown durable responses in a subset of B cell malignancies and are currently evaluated in phase II/III trials [63,64].

## 7. PI3K-Independent Modes of AKT Activation in Cancers

Mechanisms of AKT activation have been explicitly characterized and involve membrane translocation induced by the formation of PIP3, followed by the phosphorylations on T308 and S473 by PDK1 and mTORC2 respectively. It was therefore initially assumed that the chemical inhibition of the upstream activators of AKT would consistently block its activity. Emerging evidence, however, has shown that cancer cells develop a panel of alternate mechanisms to retain AKT activity despite the presence of these inhibitors. Indeed, treatment of cancer cells with PI3K inhibitors inhibits S473 phosphorylation, which is, however, rapidly recovered [42,43]. The molecular mechanisms responsible for this observation have not been fully identified but might include a persistent low level of PIP3 that is sufficient to maintain AKT phosphorylation, a downregulation of PHLLP (PH domain and leucine rich repeat protein phosphatases), a phosphatase that directly dephosphorylates AKT S473, or phosphorylation by another kinase than mTORC2 [42,65].

Furthermore, emerging evidence support a role of RTK (receptor tyrosine kinases) in restoring AKT signaling following PI3K inhibition [66]. Indeed, treatment of cancer cell lines with the PI3K inhibitor XL147 resulted in increased HER3 expression, and blocking HER3 prevented the rebound AKT activation induced by XL147 treatment [66]. It was proposed that increased HER3 activity and expression maintain membrane-bound PI3K and some level of PIP3 sufficient to induce AKT signaling despite the presence of PI3K inhibitors. Consistent with this, immunoprecipitation studies displayed an increased association between the regulatory subunit of PI3K and HER3.

**Figure 3:**
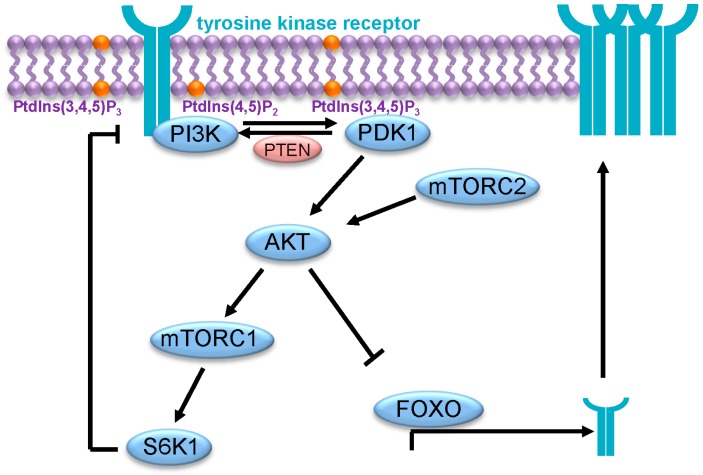
Negative feedback loops in the PI3K/AKT pathway.

The upregulation of RTK following inhibition of the PI3K/AKT pathway is part of negative feedback loops intended to limit the activation signals transmitted through the pathway (Figure 3). Therefore, removal of these loops by the pathway inhibitors blocks this regulatory process, resulting in continuous pathway activation. An important feedback loop involves the inhibition of PI3K by mTORC1/S6K1 (p70 ribosomal protein S6 kinase 1), which occurs at different levels, including inhibition of IGF1R and PDGFR (platelet derived growth factor receptors) [67,68,69,70]. Another feedback loop involves the control of RTK expression by FOXO-dependent transcriptional activation. Therefore, inhibition of AKT results in FOXO activation and increased RTK expression [66,71].

At least, two different negative feedback loops have been identified in the PI3K/AKT signaling pathway. Firstly, S6K1 inhibits PI3K activity by RTK at different levels. Secondly, AKT inhibits FOXO-mediated RTK expression. Of note, a transient inhibition of AKT activity has also been described in the case of isoform selective inhibitors of PI3K [72,73]. For instance, treatment of cancer cells with the PI3Kα-specific inhibitor BYL719 results in an initial decrease of PIP3 and AKT activity that is, however, rapidly restored through the activation of the PI3Kβ isoform [73]. Similarly, the selective inhibition of PI3Kβ only transiently inhibits AKT, as it relieves feedback inhibition of receptor tyrosine kinases, resulting in PI3Kα activation and rebound AKT signaling [72]. This demonstrates that cancer cells adapt to the inhibition of a specific isoform of PI3K by restoring AKT signaling via another PI3K isoform.

**Figure 4:**
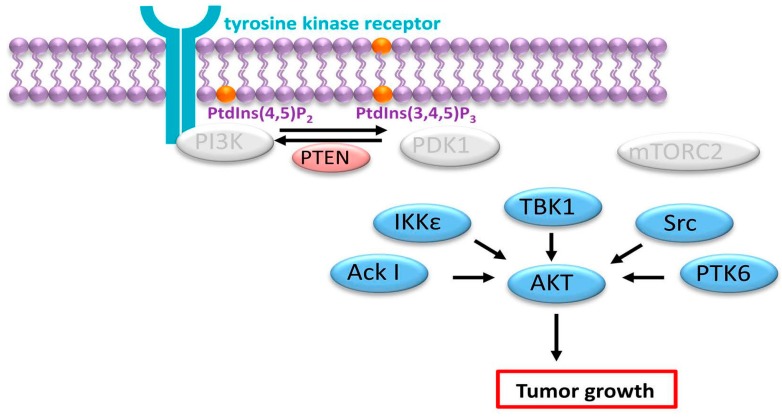
PI3K-independent AKT activation in cancer. Several kinases including Ack I, IKKε, TBK1, Src, PTK6 (protein tyrosine kinase 6) can activate AKT independently of PDK1 and mTORC2. Light gray ovals imply components of the signaling pathway that are not activated or bypassed.

In addition to the classical mode of AKT activation, recent studies have demonstrated that other kinases can interact with AKT and induce cellular transformation without requiring the PI3K signaling pathway (Figure 4) [8]. For example, the Ser/Thr kinase I-κ-B kinase epsilon (Iκκε) has the ability to activate AKT independently of the PH domain and without requiring PI3K, mTORC2 or PDK1 [74,75]. Ack I (activated CDC42-associated kinase 1), a non-receptor tyrosine kinase is also able to recruit and activate AKT by inducing the phosphorylation of the Tyr176 residue without necessitating PI3K activity [76]. Furthermore, TANK-binding kinase I (TBKI) also interacts and activates AKT in a PI3K-independent manner [77]. Finally, other kinases including protein kinase 6, Src (cellular Src kinase), DNA-PK (DNA-dependent protein kinase) and ATM (ataxia telangiectasia mutated protein) activate AKT demonstrating the complex intracellular network provided to preserve AKT activity [8].

## 8. Clinical Implications of the PI3K/AKT Unfaithful Partnership in Cancer

In view of the complex relationship between PI3K and AKT in cancers, one might speculate that PI3K inhibitors will only transiently or insufficiently block AKT activity. Several obstacles have to be overcome in order to circumstantiate this hypothesis in patients. Firstly, what read-out should be used to measure AKT activity? Indeed, whereas the phosphorylation of S473 is frequently used to assess AKT activity, recent findings have demonstrated that AKT activity is still present despite the loss of S473 phosphorylation [78]. In addition, as mentioned above, other phosphorylated amino acids of AKT are likewise involved in AKT activity independently of S473 or T308. Secondly, the effects of PI3K inhibitors on AKT activity may vary over time as cancer cells may require time to restore AKT activity following exposure to PI3K inhibitors. In this case serial tumor biopsies of cancer patients treated with PI3K inhibitors would be required which seems ethically not justifiable. Thirdly, one might raise the question if the concentrations of PI3K inhibitors used in patients are sufficient to block PI3K activity in tumors. The majority of the pharmacological analyses of PI3K inhibitors are performed on peripheral blood mononuclear cells, which do not reflect the complexity of a tumor. Therefore, without knowing whether PI3K is effectively inhibited in tumors, no clear conclusions can be drawn regarding the effects of PI3K inhibitors on AKT activity.

To date, despite major efforts in developing and testing PI3K inhibitors in clinical trial, the isoform-selective PI3K inhibitor CAL-101 (idelalisib, GS1101), targeting p110δ in the treatment of chronic lymphocytic leukemia, small lymphocytic lymphoma and follicular lymphoma is the only FDA approved agent and only few other agents have reached phase III trial stage (Supplementary Table S1). One key lesson drawn from targeted therapies used in clinical trials is the emergence of acquired resistance to those agents by cancer cells as well as by the tumor microenvironment [4]. Therefore, one can assume that targeted therapies used alone will not cure patients with advanced cancer and at least combined therapies are needed to produce stronger anticancer effects. In the case of PI3K inhibitors, prevailing studies start to reveal the resistance mechanisms employed by cancer cells against these inhibitors. For example, mutations of p110α that confer resistance to PI3K inhibitors have been identified. In addition, in breast cancer, IL-8 (interleukin-8) secretion following JAK2/STAT5 (Janus kinase 2/signal transducer and activator of transcription 5) activation has also been shown to counteract the anticancer efficacy of PI3K inhibitors [42]. Likewise, in breast cancer, an upregulation of HER3 (human epidermal growth factor receptor 3) following PI3K inhibition also attenuates the response of cancer cells to these inhibitors [79]. Finally, in lymphoma, overexpression of PAK1 (p21-activated kinase 1) was shown to mediate resistance to PI3K inhibition [80]. Thus cancer cells develop resistance mechanisms to PI3K inhibitors that are important to identify in order to design new therapeutic strategies that will overcome these resistances and improve the anticancer efficacy of PI3K inhibitors.

As described here, another level of complexity is present in the pathway as PI3K-independent mechanisms of activation of AKT are used by cancer cells. As a consequence, PI3K inhibitors do not persistently inhibit AKT signaling and therefore a rationale exists to combine PI3K and AKT inhibitors in cancer therapy. Consistently, *in vitro* experiments showed that the anti-proliferative effects of PI3K inhibitors are enforced by AKT inhibitors [43]. Furthermore, it was demonstrated that AKT inhibitors induce the overexpression of growth factors following the loss of a negative feedback loop [71]. In turn, increased expression of growth factors augments PI3K activity that reduces the efficacy of AKT inhibitors.

## 9. Conclusions

Although multiple studies have demonstrated that AKT is a major downstream effector of PI3K, recent evidence outlines the importance of AKT and PI3K functioning independently of one another in cancer. As a consequence, PI3K inhibitors might have a limited effect on AKT activity. Future studies are needed to further characterize the complex relationship between PI3K and AKT in cancer. In addition, combining PI3K and AKT inhibitors in cancer therapy warrants further investigation.

## Supplementary Materials

Supplementary materials can be found at http://www.mdpi.com/1422-0067/16/09/21138/s1.
