# Supplementary material for: PI3K and AKT: Unfaithful Partners in Cancer
Source: Int J Mol Sci. 2015 Sep 3;16(9):21138–52. doi: 10.3390/ijms160921138 (PMC4613246; doi:10.3390/ijms160921138)
Supplement: Supplementary file 1 [file ijms-16-21138-s001.pdf]

Supplementary Information

Table S1. PI3K and AKT inhibitors and their clinical development. Inhibitors of the PI3 kinase and AKT are listed with emphasis on their molecular target, mechanism of action and clinical trial stage (as according to ClinicalTrial.gov August 2015). CAL-101 (idelalisib, GS1101) was FDA approved in 2014 for the treatment of CLL (chronic lymphocytic leukemia), SLL (small lymphocytic lymphoma) and follicular lymphoma. FDA granted orphan drug designation to IPI145 (INK1197, duvelisib) for the potential treatment of CLL and SLL. No further PI3K or AKT inhibitor has reached US FDA approval by August 2015.

Agent	Molecular Target	Mechanism of Action	Clinical Trial Stage
Pan-PI3K Inhibitors			
BAY80-6946 (copanlisib)	Class I PI3Ks (preferentially p110 alpha/delta)	inhibits catalytic activity of Class I PI3Ks, preferentially p110 alpha and delta	Phase III
BKM120 (buparlisib)	Class I PI3Ks	ATP-competitive mechanism by binding to ATP-binding site of the lipid kinase of Class I PI3K isoforms, G2-M arrest upon binding to tubulin and microtubule destabilization	Phase III
GDC0941 (pictilisib)	Class I PI3Ks	ATP-competitive inhibitor	Phase II
PX-866	Class I PI3Ks	irreversible inhibition of PI3K by interaction with the ATP catalytic site	Phase II
XL147 (pilaralisib, SAR245408)	Class I PI3Ks	ATP-competitive inhibitor	Phase II
CH5132799	Class I PI3Ks (preferentially p110 alpha)	ATP-competitive inhibitor	Phase I
CUDC-907	PI3K and HDAC (histone deacetylases)	inhibition of PI3K and HDAC activity	Phase I
ZSTK474	Class I PI3Ks (preferentially p110 delta)	ATP-competitive inhibitor	Phase I
WX037	Class I PI3Ks	no further information	Phase I **
AMG511, BAG956, NVP-QAV-572, PI 828, PIK-90, PF-4989216, TG100713, wortmannin			no clinical cancer trial registered

Table S1. *Cont.*

Agent	Molecular Target	Mechanism of Action	Clinical Trial Stage
Dual pan-PI3K/mTOR Inhibitors			
BEZ235 (dactolisib)	PI3Ks and mTOR	ATP-competitive inhibitor	Phase II
BGT226	PI3Ks and mTOR	ATP-competitive inhibitor	Phase II
GDC0980 (apitolisib, RG7422)	PI3Ks and mTOR	ATP-competitive inhibitor	Phase II
LY3023414	PI3Ks and mTOR	ATP-competitive inhibitor	Phase II
PF-05212384 (gedatolisib, PKI587)	PI3Ks and mTOR	ATP-competitive inhibitor	Phase II
XL765 (SAR245409, voxtalisib)	PI3Ks and mTOR	ATP-competitive inhibitor	Phase II
PF04691502	PI3Ks and mTOR	ATP-competitive inhibitor	Phase II ***
DS7423	PI3Ks and mTOR	inhibits PI3K and mTOR, induces p53-dependent apoptosis	Phase I
GDC0084	PI3Ks and mTOR	ATP-competitive inhibitor	Phase I
GSK2126458 (omipalisib, GSK458)	PI3Ks and mTOR	ATP-competitive inhibitor	Phase I
PWT33597	PI3Ks and mTOR	ATP-competitive inhibitor	Phase I
SF1126 (LY294002/SF1101)	PI3Ks and mTOR	ATP-competitive inhibitor	Phase I
VS-5584 (SB2343)	PI3Ks and mTOR	ATP-competitive inhibitor	Phase I
GSK1059615	PI3Ks and mTOR	ATP-competitive inhibitor	Phase I **
GNE-317, GNE-477, PI-103, PI-3065, PKI-402			no clinical cancer trial registered

Table S1. *Cont.*

Agent	Molecular Target	Mechanism of Action	Clinical Trial Stage
Isoform-Selective PI3K Inhibitors			
CAL-101 (idelalisib, GS1101)	p110 delta	ATP-competitive inhibitor	FDA approved 2014<br>Phase III
IPI145 (INK1197, duvelisib)	p110 delta/gamma	ATP-competitive inhibitor	FDA granted orphan drug designation<br>Phase III
GDC0032	p110 alpha/delta/gamma	ATP-competitive inhibitor	Phase III
BYL719 (alpelisib)	p110 alpha	ATP-competitive inhibitor	Phase II
INCB040093	p110 delta	no further information	Phase II
AMG 319	p110 delta	no further information	Phase I
AZD8186	p110 beta/delta	interaction with kinase activity	Phase I
AZD8835	p110 alpha/delta	interaction with kinase activity	Phase I
GS-9820 (acalisib)	p110 delta	interaction with kinase activity	Phase I
GSK2636771	p110 beta	interaction with kinase activity	Phase I
MLN1117 (INK1117)	p110 alpha	ATP-competitive inhibitor	Phase I
PWT-143	p110 delta	no further information	Phase I
RP6530	p110 delta/gamma	no further information	Phase I
SAR260301	p110 beta	interaction with kinase activity	Phase I
A66, AS-252424, AS-604850, AS-605240, CAL-130, CAL-263, CAY10505, CZC24832, GNE-293, HS-173, IC-87114, IPI443, KAR4139, KIN193 (AZD6482), PI3Kgamma inhibitor 1, PIK-75, PIK-293, PIK-294, PK-93, RV1729, TG100-115, TGX-221, TRG1202			no clinical cancer trial registered

Table S1. *Cont.*

Agent	Molecular Target	Mechanism of Action	Clinical Trial Stage
AKT Inhibitors			
LY317615 (enzasturin)	PKCbeta, AKT, GSK3beta and ribosomal protein S6	inhibits phosphorylation of PKCbeta, AKT, GSK3beta and ribosomal protein S6 by binding to ATP-binding site	Phase III
perifosine (KRX-0401)	AKT interaction with phospholipids	Inhibition of PH domain mediated membrane recruitment of AKT results in reduced AKT phosphorylation	Phase III *
AZD5363	AKT1, 2, 3	ATP-competitive inhibitor	Phase II
GDC0068 (ipatasertib)	AKT1, 2, 3	ATP-competitive inhibitor	Phase II
GSK2110183 (afuresertib)	AKT1, 2, 4	ATP-competitive inhibitor	Phase II
GSK2141795 (GSK795, uprosertib)	AKT1, 2, 3	ATP-competitive inhibitor	Phase II
LY2780301	p70S6 kinase and AKT	ATP-competitive inhibitor of p70S6 kinase and AKT	Phase II
MK-2206	AKT1, 2, 3	allosteric inhibitor	Phase II
ONC201 (TIC10)	AKT, ERK (extracellular signal regulated kinases)	inactivation of AKT and ERK	Phase II
PBI-05204 (oleandrin)	AKT, FGF-2 (fibroblast growth factor 2), NF-κB and p70S6K	inhibition of Na-K ATPase pump activity	Phase II
RX-0201	AKT1	AKT1 antisense oligonucleotide that binds to mRNA and inhibits translation	Phase II
triciribine (PTX-200)	AKT1, 2, 3	inhibits AKT phosphorylation and AKT kinase activity, DNA synthesis inhibitor	Phase II
AT13148	AGC kinases including AKT, p70S6, PKA, SGK and Rho	multiple AGC kinase inhibitor	Phase I
GSK690693	AKT1, 2, 3	ATP-competitive inhibitor	Phase I **
10-DEBC, 3,5-dimethyl PIT-1, 3CAI (AKT Inhibitor XIX), A-443654, A-674563, AKTi-1/2, AKT inhibitor VIII, AT7867, CCT128930, deguelin, erucylphosphocholine, FPA 124, KP372-1, palomid 529, PHT-427, SC 66			no clinical cancer trial registered

* one phase III trial (colorectal cancer) completed, one phase III trial (multiple myeloma) discontinued after interim results, several phase II trials active or completed; ** study terminated; *** three phase II trials terminated due to tolerability findings, clinical development discontinued in 2012.

Table S2. Genetic alterations in the PI3Ks/AKT signaling pathway in human cancers. Genetic alterations of the PI3K, PTEN and AKT resulting in an overactivation of the PI3K/AKT signaling pathway and manifestation of human cancers are listed. Cancer types with a frequency of >5% of the corresponding genetic alteration are displayed.

**Genetic Alteration**	**Cancer Type**
Class IA PI3K—PIK3CA	
Activating mutation	Endometrial, Breast, Ovarian, Colorectal, Bladder, Lung, Cervical, Glioblastoma, Head and neck, Oesophageal, Melanoma, Hepatocellular, Gastric, Pancreatic
Copy number gain/Amplification	Head and neck, Cervical, Lung, Lymphoma, Ovarian, Gastric, Thyroid, Prostate, Breast, Glioblastoma, Endometrial, Oesophageal, Leukemia
Increased expression	Prostate
Class IA PI3K—PIK3CB	
Copy number gain/Amplification	Lung, Thyroid, Ovarian, Lymphoma, Glioblastoma, Breast
Increased expression	Prostate, Bladder, Colorectal
Class IA PI3K—PIK3CD	
Copy number gain	Glioblastoma
Increased expression	Neuroblastoma, Glioblastoma
Class IA PI3K—PIK3R1	
Inactivating mutation	Endometrial, Pancreatic, Glioblastoma, Colorectal
Copy number loss	Ovarian
Decreased expression	Prostate, Breast, Lung, Ovarian, Bladder
Class IA PI3K—PIK3R2	
Amplification	Lymphoma
Increased expression	Colorectal, Breast
Class IA PI3K—PIK3R3	
Copy number gain	Ovarian
Class IB PI3K—PIK3CG	
Copy number gain	Ovarian
Increased expression	Breast, Prostate, Medulloblastoma

Table S2. *Cont.*

Class IB PI3K—PIK3R5	
Activating mutation	Melanoma
PTEN	
Loss of heterozygosity	Glioblastoma, Gastric, Melanoma, Prostate, Breast, Endometrial, Leukemia
Inactivating mutation	Glioblastoma, Melanoma, Prostate, Endometrial, Colorectal, Ovarian, Breast, Leukemia, Gastric, Hepatocellular, Renal, Vulva, Bladder, Lung, Thyroid
Decreased expression	Breast, Melanoma, Prostate
AKT1	
Amplification	Gastric
AKT2	
Amplification	Head and neck, Pancreatic, Ovarian
